# Memory Alteration Test to Detect Amnestic Mild Cognitive Impairment and Early Alzheimer’s Dementia in Population with Low Educational Level

**DOI:** 10.3389/fnagi.2017.00278

**Published:** 2017-08-22

**Authors:** Nilton Custodio, David Lira, Eder Herrera-Perez, Rosa Montesinos, Sheila Castro-Suarez, José Cuenca-Alfaro, Lucía Valeriano-Lorenzo

**Affiliations:** ^1^Servicio de Neurología, Instituto Peruano de Neurociencias Lima, Peru; ^2^Unidad de Diagnóstico de Deterioro Cognitivo y Prevención de Demencia, Clínica Internacional Lima, Peru; ^3^Unidad de Investigación, Instituto Peruano de Neurociencias Lima, Peru; ^4^GESID Lima, Peru; ^5^Instituto Nacional de Salud del Niño Lima, Peru; ^6^Servicio de Medicina de Rehabilitación, Instituto Peruano de Neurociencias Lima, Peru; ^7^Servicio de Neurología de la Conducta, Instituto Nacional de Ciencias Neurológicas Lima, Peru; ^8^Unidad de Neuropsicología, Instituto Peruano de Neurociencias Lima, Peru

**Keywords:** memory alteration test, mild cognitive impairment, dementia, Alzheimer’s disease, neuropsychological assessment, validity and reliability, diagnostic test accuracy

## Abstract

**Background/Aims**: Short tests to early detection of the cognitive impairment are necessary in primary care setting, particularly in populations with low educational level. The aim of this study was to assess the performance of Memory Alteration Test (M@T) to discriminate controls, patients with amnestic Mild Cognitive Impairment (aMCI) and patients with early Alzheimer’s Dementia (AD) in a sample of individuals with low level of education.

**Methods**: Cross-sectional study to assess the performance of the M@T (study test), compared to the neuropsychological evaluation (gold standard test) scores in 247 elderly subjects with low education level from Lima-Peru. The cognitive evaluation included three sequential stages: (1) screening (to detect cases with cognitive impairment); (2) nosological diagnosis (to determinate specific disease); and (3) classification (to differentiate disease subtypes). The subjects with negative results for all stages were considered as cognitively normal (controls). The test performance was assessed by means of area under the receiver operating characteristic (ROC) curve. We calculated validity measures (sensitivity, specificity and correctly classified percentage), the internal consistency (Cronbach’s alpha coefficient), and concurrent validity (Pearson’s ratio coefficient between the M@T and Clinical Dementia Rating (CDR) scores).

**Results**: The Cronbach’s alpha coefficient was 0.79 and Pearson’s ratio coefficient was 0.79 (*p* < 0.01). The AUC of M@T to discriminate between early AD and aMCI was 99.60% (sensitivity = 100.00%, specificity = 97.53% and correctly classified = 98.41%) and to discriminate between aMCI and controls was 99.56% (sensitivity = 99.17%, specificity = 91.11%, and correctly classified = 96.99%).

**Conclusions**: The M@T is a short test with a good performance to discriminate controls, aMCI and early AD in individuals with low level of education from urban settings.

## Introduction

Mild cognitive impairment (MCI) is a well recognized risk factor for Alzheimer’s disease (AD), and for the predemential phase of this and other dementias (Albert et al., 2011; Li et al., 2011; Cooper et al., 2015). The need for research aimed to AD early diagnosis have been highlighted in several studies directed towards the prevention and control of the worldwide progression of the disease (Richard et al., 2012; Barnett et al., 2013). Thus, it is necessary to have brief and reliable instruments to early diagnosis in primary care settings (Custodio et al., 2017).

Globally, there is a generalized low detection of dementia in the community. This is a real challenge in Latin America (LA; Lang et al., 2017), where previous studies showed that the majority of medical doctors perceive that their practices for diagnosis and treatment of dementia are inadequate, underscoring that this deficiency is higher in general practitioners than in specialists (Olavarria et al., 2015). In addition, other challenge in LA countries is the lack of validated and standardized instruments to assess cognition and functionality in indigenous populations, in rural areas, with a language other than Spanish, or with low levels of education (Maestre, 2012; Parra, 2014).

Various instruments have been developed to detect dementia (Folstein et al., 1975; Mattis, 1976; Roth et al., 1986), but there is not still gold standard short test. The Mini-Mental State Examination (MMSE), the most widely used short test, is especially inadequate in less-educated populations (Rosselli et al., 2000; Scazufca et al., 2009) because its low validity and diagnostic accuracy in this populations (Lonie et al., 2009; Mitchell, 2009; Carnero-Pardo et al., 2011b). Other short tests include task that require reading and writing abilities or involve the use of pencil and paper, which affects its use in populations with a low educational level (Carnero-Pardo et al., 2011a).

In Peru, several short tests have been validated in urban samples from Lima, including the clock drawing test (CDT)—Mano’s version (Custodio et al., 2011), the Addenbrooke’s cognitive examination (ACE; Custodio et al., 2011), the memory alteration test (M@T; Custodio et al., 2014), the INECO frontal screening (IFS; Custodio et al., 2016b) and the Peruvian version of the Eurotest (Oscanoa et al., 2016). However, neither of these tests were validated in LA low-educated populations (Paddick et al., 2017).

The M@T is a short cognitive test to detect dementia, able to discriminate between controls, patients with amnestic MCI (aMCI), and patients with early AD (Rami et al., 2007, 2010; Custodio et al., 2014; Ozer et al., 2016). It has been reported the utility of M@T in patients with low level of education (Sousa et al., 2015), however, validation studies of short cognitive tests for detecting aMCI and AD in population with low-level education are scarce (Paddick et al., 2017). Thus, the aim of the present study is to assess the validity of M@T to discriminate between controls, patients with aMCI and patients with early AD in a sample of individuals with low level of education.

## Materials and Methods

### Design of the Study

Diagnostic test cross-sectional study to evaluate the performance of the M@T (study test), compared to the neuropsychological evaluation (gold standard test).

#### The Study Test

The M@T is a valid screening test that assess the temporal orientation and different types of memory (episodic, textual and semantic) and discriminates between healthy elderly subjects, patients with aMCI and patients with early AD. This is a cognitive test with high internal consistency and validity, short application (5–10 min), easy to perform and to interpret, developed in Spain (Rami et al., 2007) and validated in Peru (Custodio et al., 2014). Its results are mildly influenced by educational level, thereby the cutoff points are 36/37 and 37/38 for subjects with <8 years and ≥8 years of education, respectively (Carnero-Pardo et al., 2011a).

This test is totally oral and do not require reading or writing skills or the use of pencil and paper, allowing the evaluation of very low educated subjects. All the questions of M@T have a single correct answers, and covering five domains: temporal orientation (5), short term memory (10), semantic memory (15), free recall (10) and facilitated recall (10). Thus, the maximum score of this test is 50 points.

#### The Gold Standard Test

The neuropsychological assessment is the detailed evaluation of the cognitive functions, by means of a neuropsychological battery adapted to Peruvian population. The battery included the following tests: Rey Auditory Verbal Learning Test (RAVLT; Rey, 1941), Logical Memory—Subtest of Wechsler Memory Scale Revised (Wechsler, 1997), Trail Making Test A and B (Partington and Leiter, 1949), Rey-Osterrieth Complex Figure Test (ROCF; Rey, 1941), Boston Naming Test (Kaplan et al., 1983), Wisconsin Card Sorting Test (WCST; Nelson, 1976), Letter-Number and Digit Span, subtests of Wechsler Adult Intelligent Scale III (Wechsler, 1997).

Following the order of the tests mentioned above, the neuropsychological battery has the main purpose to explore cognitive skills such as verbal memory and verbal learning through retention and evocation of verbal stimuli, immediate recall and delayed recall of stories, scanning and visuomotor tracking, divided attention, cognitive flexibility, visual memory and visuospatial construction skill. Also it appraises language skills like naming ability and word retrieval, executive functioning like forming concepts, conceptual flexibility as well attentional control, working memory and span of immediate verbal recall.

The decision criterion is two standard deviations below the mean in order to establish deficit in the cognitive domain assessed. These values were collected from the original articles for each selected test. Throughout the study, the neuropsychologists were blinded to results of M@T.

#### Population and Sample

The study was carried out in elderly care home centers of two districts of Lima (four from “Carabayllo” and two from “Cercado de Lima”) between March and September of 2015. We included subjects older than 60 years, Spanish speakers with low educational level (<4 years of completed formal education), excluding those with any condition that might cause cognitive impairment non-related to neurodegenerative etiology (history of substances addiction or abuse, depression, hypothyroidism, vitamin B12 deficiency, chronic hepatopathy or nephropathy, neuroinfections by HIV or syphilis, severe brain injury, sub-dural hematoma, cerebrovascular illness, vascular dementia suggestion (Hachinski Ischemic Score >4), etc.) or that could affect their performance to realize the cognitive tests (auditory, visual or other physical deficits).

Additionally, we excluded to patients that consumed any of following drugs: opioid analgesics, decongestants, anti-spasmodics, anti-cholinergics, anti-depressants, antiarrhythmics, antipsychotics, anti-emetics, anxiolytics and valproate.

### Procedures

We requested the list of regular users (i.e., assistance frequency >3 times/week) of the elderly care home centers. By means of simple random sampling (table of random numbers), the potential participants were selected until completing a quota of half of available population (sample size = 0.5 N), consented to participate, and provided information necessary to assess compliance with eligibility criteria. The evaluation of cognitive impairment was performed in three successive stages: (1) screening (to detect cases with cognitive impairment); (2) nosological diagnosis (to determinate specific disease that is the cause of cognitive impairment); and (3) final classification (to differentiate disease subtypes).

In the screening phase, an integral clinical evaluation was performed, including measurement of anthropometry and blood pressure, application of Pfeffer Functional Activities Questionnaire (PFAQ) and cognitive screening tests (MMSE and CDT). If any cognitive test was positive for impairment, it was repeated by a different evaluator. The confirmed cases were considered as patients with cognitive impairment (PCI). According to educational level, the cutoff score used was 23 for subjects with 4 years of education, 21 for subjects with 1–3 years of education, and 18 for subjects with less than 1 year of education (Custodio and Lira, 2014). The MMSE and CDT was applied to study subjects, and PFAQ was applied to their caregivers/accompanist.

In the second stage, the PCIs were assessed using blood tests (hemogram, glucose, electrolytes, transaminases, rapid plasma reagin (RPR), urea, creatinine, vitamin B12, folic acid, free T3 and T4, and ultra-sensitive TSH), images studies (brain tomography and/or magnetic resonance imaging), and Beck Depression Inventory II (BDI-II) for discarding non-neurodegenerative causes of cognitive impairment. We applied the DSM-IV (American Psychiatric Association, 2000) criteria to diagnosis dementia, and the Clinical Dementia Rating (CDR; Hughes et al., 1982) for staging dementia. The CDR was applied to both study subjects and caregivers/accompanist.

Finally, in the third stage, we performed the neuropsychological evaluation of patients with MCI or dementia to typify its subtype. We applied the criteria of Petersen (Petersen et al., 1999) and NINCDS-ADRDA (McKhann et al., 1984) to classify as aMCI or AD, respectively. The doubtful cases (regarding typification) were resolved by researchers consensus.

The subjects with negative results in all tests for cognitive assessment were considered as cognitively normal (controls). The M@T was applied to study subjects in first stage and the evaluators were blinded to the results of this psychometric. The results of M@T were not used as part of the neuropsychological battery for diagnosis. The team of evaluators of the second and third phases (expert neurologists and neuropsichologists) was different from the team of the first phase (students of medicine and psychology supervised by expert neurologists).

### Statistical Methods

The corresponding descriptive statistics were performed. The analysis was performed comparing the cognitive groups (controls, aMCI and AD) by pairs. For this purpose we applied *T* tests (for quantitative variables) and Chi Square (for categorical variables). We assessed the internal consistency (Cronbach’s alpha coefficient) and the concurrent validity (Pearson’s ratio coefficient between the M@T and CDR scores).

We performed a logistic regression (logit) for each pair of study groups (early AD/aMCI, aMCI/control, and early AD/control), using a model of two variables: final diagnosis as dependent variable and test as independent variable. We applied postestimation analysis to compute area under receiver operating characteristics (ROC) curve and graph ROC curve, and calculate validity measures (sensitivity, specificity and positive and negative predictive values).

Additionally, we calculated the diagnostic accuracy (percentage of correctly classified individuals) for M@T, MMSE and CDT. The maximum values of this measure were the standard for the cut-off scores selection of sensitivity, specificity and predictive values. Finally, we compared the AUC of this tests using the method of Hanley and McNeil. The tests were performed at 95% confidence using the STATA software (version 12.0).

### Ethical Aspects

This study was carried out in accordance with the recommendations of the Council for International Organizations and Medical Sciences (CIOMS). A written informed consent was obtained from all participants or their carers in accordance with the Declaration of Helsinki. The protocol was approved by the Ethics Committee of the *Universidad de San Martin de Porres*.

## Results

### Flow of Participants

The first stage started with 346 participants, but 41 were missed (14 due to withdrawal of informed consent, 21 due to difficulty in attending scheduled appointments and six due to caregiver or evaluator illness). In the second stage, 22 of 305 participants were missed (seven due to difficulty in attending scheduled appointments, four for lack of blood tests results and 11 for lack of brain tomography).

Finally, 283 participants completed the third stage. However, 36 participants were not included in the present analysis because they were classified as non-amnesic MCI (16), vascular dementia (6), Frontotemporal dementia (4), dementia associated with Parkinson’s disease (2) and other unspecified dementias (8).

### Data of Participants

**Figure 1:**
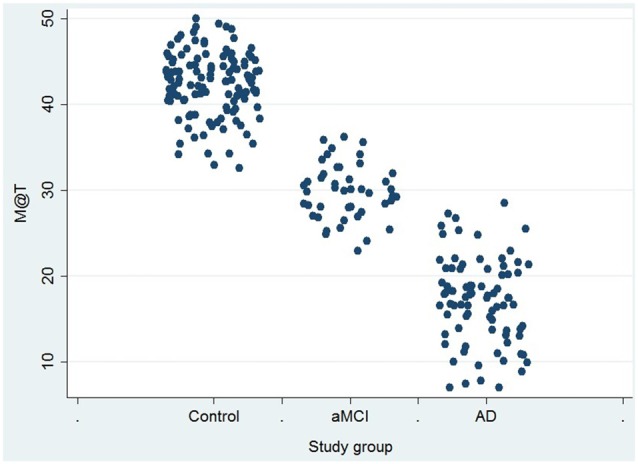
Score in Memory Alteration Test (M@T) in 247 low-level education individuals from Lima-Peru, according to definitive diagnosis. AD, Alzheimer’s dementia; aMCI, amnestic mild cognitive impairment.

Statistical analysis of the sociodemographic data, MMSE scores and M@T scores were performed according to the comparison groups. In patients with AD, compared to those with aMCI, age was significantly higher and test scores (MMSE, CDT and M@T) were significantly lower. On the other hand, in the patients with aMCI the age was significantly higher and the M@T and CDT scores were significantly lower, compared to control subjects (Table 1). The M@T and CDT scores showed a differential distribution according to the comparison group, behaving as a trend (Figure 1). The results of the neuropsychological assessment are detailed in Table 2.

**Table 1:** Demographic characteristics and cognitive test scores in 247 low-level education individuals from Lima-Peru, according to definitive diagnosis.

	Study group				
	Early Alzheimer’s dementia (*n* = 81)	amnestic mild cognitive impairment (*n* = 45)	Control (*n* = 121)	*p*-value 1^†^ (early AD vs. aMCI)	*p*-value 2^‡^ (aMCI vs. control)
Sex: female	52 (64.20%)	30 (66.67%)	68 (56.20%)	0.781	0.223
Age, years^§^	74.18 (3.81)	71.09 (4.20)	69.53 (4.11)	0.000**	0.032*
Education, years^§^	2.65 (1.28)	2.53 (1.46)	2.57 (1.45)	0.629	0.885
MMSE, score^§^	18.32 (2.78)	21.36 (0.98)	22.02 (1.26)	0.000**	0.056
CDT, score^§^	2.42 (1.69)	8.02 (1.06)	8.75 (0.91)	0.000**	0.000**
M@T, score^§^	17.54 (4.67)	30.53 (2.54)	41.97 (2.68)	0.000**	0.000**

AD, Alzheimer’s dementia; aMCI, amnestic mild cognitive impairment; MMSE, Mini Mental State of Examination; CDT, Clock Drawing Test—Mano’s version; M@T, Memory Alteration Test; ^§^Data showed as mean (standard deviation); ^†^p-value for comparation between early AD and aMCI; ^‡^p-value for comparation between aMCI and control; *p-value < 0.05; **p-value < 0.001.

**Table 2:** Results of the neuropsychological assessment in 247 low-level education individuals from Lima-Peru, according to definitive diagnosis.

Test	Sub-test	Study group Test		
		Early Alzheimer’s dementia (*n* = 81)	amnestic mild cognitive impairment (*n* = 45)	Control (*n* = 121)
RAVLT	Free-recall	3.22 (0.72)	5.33 (0.67)	6.17 (0.90)
	Recognition	5.84 (1.01)	9.93 (0.98)	13.02 (1.05)
Logical memory	Immediate recall	1.89 (1.07)	6.51 (0.94)	12.00 (1.38)
	Delayed recall	1.42 (0.91)	6.18 (0.74)	11.83 (1.24)
Trail making test	Test A (s)	80.93 (8.30)	67.96 (7.80)	54.00 (8.74)
	Test B (s)	188.98 (21.37)	115.09 (12.39)	99.31 (14.92)
ROCF	Copy	16.62 (2.29)	24.93 (2.23)	28.35 (1.93)
	Recall	6.30 (1.65)	9.67 (1.72)	13.97 (2.81)
Test of denomination of Boston		13.85 (3.70)	28.67 (5.44)	51.59 (3.37)
WCST	Categories	2.73 (0.63)	4.16 (0.64)	4.97 (0.53)
	Perseverations	13.07 (2.76)	6.13 (1.79)	1.86 (0.73)
Letter—Number		4.83 (0.75)	7.13 (0.94)	10.10 (1.66)
Digit span		2.40 (0.72)	4.24 (0.43)	4.90 (0.55)

*RAVLT, Rey Auditory Verbal Learning Test; ROCF, Rey-Osterrieth Complex Figure; WCST, Wisconsin Card Sorting Test*.

### Psychometric Properties of M@T

Internal consistency (Cronbach’s alpha coefficient: 0.79) and concurrent validity (*r* = 0.79; *p* < 0.01) were good. In relation to the M@T cutoff, a score of 26 allows to discriminate between early AD and aMCI (sensitivity = 100.00% and specificity = 97.53%), with an accuracy of 98.41%. Similarly, a score of 35 allows discriminating between aMCI and controls (sensitivity = 99.17% and specificity = 91.11%), with an accuracy of 96.99% (Table 3).

**Table 3:** Cut-off points and diagnostic performance of M@T and MMSE to discriminate between AD, aMCI.

	Discrimination between early AD and aMCI			Discrimination between aMCI and controls			Discrimination between early AD and controls		
	M@T	MMSE	CDT	M@T	MMSE	CDT	M@T	MMSE	CDT
Optimal cut-off ^§^	26	21	5	35	21	8	29	21	5
Sensitivity	100.00	86.67	100.00	99.17	90.91	95.04	100.00	90.91	100.00
Specificity	97.53	75.31	87.65	91.11	13.33	31.11	98.77	75.31	87.65
Correctly classified (%)	98.41	79.37	92.06	96.99	69.88	77.71	99.50	84.65	95.05
Likelihood ratio +	40.500	3.51	8.10	11.16	1.05	1.38	81.00	3.68	8.10
Likelihood ratio −	0.000	2.07	0.00	0.009	0.68	0.16	0.000	0.12	0.00
Area under	0.9960^†^	0.8278	1.0000	0.9956^†‡^	0.6536	0.6869	1.0000^†^	0.8820	1.0000
curve [95% CI]	[0.99–1.00]	[0.76–0.90]	[1.00–1.00]	[0.99–1.00]	[0.57–0.74]	[0.60–0.78]	[1.00–1.00]	[0.83–0.93]	[1.00–1.00]

*AD, Alzheimer’s dementia; aMCI, amnestic mild cognitive impairment; MMSE, Mini Mental State of Examination; M@T, Memory Alteration Test; CI, Confidence interval; ^§^Cut-off base on maximum value of correctly classified. ^†^Significant difference regarding the MMSE (p-value < 0.05); ^‡^Significant difference regarding the CDT (p-value < 0.05)*.

**Figure 2:**
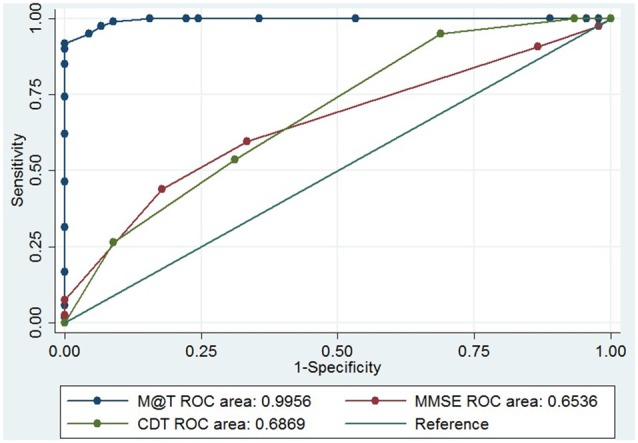
Receiver operating characteristics (ROC) curve of M@T, MMSE and CDT to discriminate between patients with aMCI and controls in 166 low-level education individuals from Lima-Peru. MMSE, Mini Mental State of Examination; CDT, Clock Drawing Test—Mano’s version; M@T, Memory Alteration Test.

**Figure 3:**
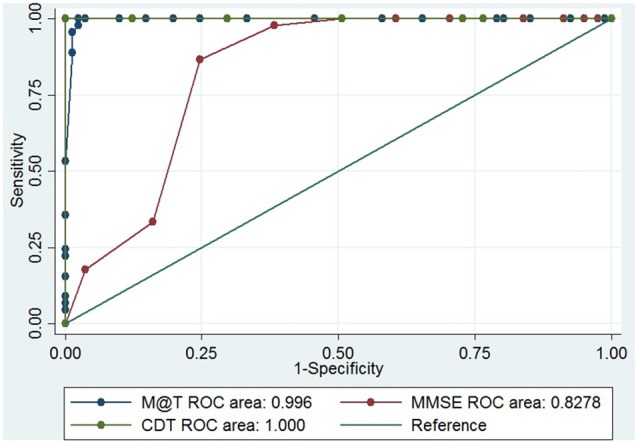
ROC curve of M@T, MMSE and CDT to discriminate between patients with aMCI and early AD in 126 low-level education individuals from Lima-Peru. MMSE, Mini Mental State of Examination; CDT, Clock Drawing Test—Mano’s version; M@T, Memory Alteration Test.

The performance of the M@T to discriminate between early AD and aMCI was 0.9960 (Figure 2) and to discriminate between aMCI and controls was 0.9956 (Figure 3). The discriminatory performance of M@T was significantly higher than the MMSE (*p* = 0.000) for all combinations of analyzed group pairs. Furthermore, the performance of M@T was significantly higher than CDT to discriminate between patients with aMCI from controls (Table 3). Additionally, we performed an analysis for assessing if the score M@T is statistically associated with clinical diagnosis (early AD or aMCI; Supplementary Table S1).

## Discussion

### Implications

This study shows a good performance of M@T to discriminate between early AD and aMCI in subjects with less than 4 years of education. These results are similar to those previously obtained with a sample of 6.5 years of average education (AUC: 0.9986; Custodio et al., 2014) and slightly higher than those obtained in a Spanish sample with 8 years of average education (AUC: 0.9300; Rami et al., 2007).

Similarly, we found a good performance to discriminate between patients with aMCI and controls (AUC: 0.9956), which was slightly lower than that reported previously (AUC: 0.9986; Custodio et al., 2014), but also higher than that obtained in a Spanish sample (AUC: 0.932; Rami et al., 2007). Our research has also shown a good correlation coefficient between M@T and MMSE, which suggests convergent validity. This is a finding similar to that previously obtained with the Portuguese version of the M@T (Sousa et al., 2015).

Additionally, we found that the performance of M@T is higher than MMSE and CDT for discriminating both AD vs. aMCI and aMCI vs. controls. This findings can be explained because M@T evaluates episodic and semantic memory, which have their biological substrate in the hippocampus, the medial temporal lobe and temporal neocortex, areas that are early affected in AD (Rami et al., 2007). In contrast, the MMSE evaluates orientation, language, praxia and general aspects of memory and the CDT evaluates planning, visuospatial and constructive functions. Thus, MMSE is not able to discriminate between AD and aMCI (Tombaugh and McIntyre, 1992; Wind et al., 1997; Rami et al., 2009), and CDT is more appropriate to detect advanced stages of AD (Custodio et al., 2016a).

According to recent UNESCO data, 16% of adults have emerged from education systems without basic literacy skills, which is a major problem in the regions of Sub-Saharan Africa and South Asia, where more than 1/3 of adults are illiterate. Around the world, at least 20 countries have adult literacy rates less than 60% and 43 countries have adult literacy rates less than 75% (UNESCO Institute for Statistics, 2016). Thus, this population constitutes an important group and their needs emerge as public health focus. In this context, valid diagnostic tests for its use in people with low educational level are required.

There are evidence about the demographic influences (e.g., age, gender, education, and residence rural/urban) on the performance of several cognitive tests (Freitas et al., 2015; Li et al., 2016; Xie et al., 2016). Particularly, the education is a key factor since dementia is under-recognized among people with low education levels (Xie et al., 2016). Thereby the international norms of MMSE, the most broadly used cognitive screening instrument, consider different optimal cut-off points depending of educational level to improve screening precision for cognitive impairment (Moraes et al., 2010; Kim et al., 2012; Freitas et al., 2015; Li et al., 2016; Xie et al., 2016). Regarding previous results in Peruvian subjects with at least 6 years of education (Custodio et al., 2014), our data showed that performance with M@T is affected by education and cut-off points should be adjusted.

Additionally, previous studies have shown that non-specialist physicians have difficulties in effectively identifying aMCI and early AD. Thus, it is necessary to develop clinically useful, non-invasive and/or cost-effective, screening tools (Connolly et al., 2011), which must be applicable in primary care centers (Laske et al., 2015)^.^ In Peru, M@T has been shown to be a reliable test with high precision to discriminate between early AD, aMCI and normal cognition in samples of low educational level (Custodio et al., 2014) and, according to the results of this study, in samples with very low educational level. There are evidence suggesting a progression between various clinical states, beginning with MCI and, after a period of up to 5 years, evolving to dementia in its various sequential stages of severity (De Meyer et al., 2010; Derby et al., 2013). Our results show that, in fact, the average age is higher among patients with AD compared to patients with aMCI and, in turn, they are older than the control subjects.

In addition to age, another important sociodemographic variable is the sex. Several population-based studies have shown nearly two-thirds of individuals diagnosed with AD are females (Dal Forno et al., 2005). In this sense, the sociodemographic profile of the patients included in this study is consistent with that previously reported in the world literature.

In our sample, MMSE and CDT showed a suboptimal performance for discriminating between aMCI and healthy controls. This findings contrasts with previous studies, which found an AUC values higher than 0.80 and 0.70 with the use of MMSE and CDT, respectively (Cacho et al., 2010; Kato et al., 2013). However, a brazilian study showed a low performance of these tests (0.63 and 0.59, respectively; Ladeira et al., 2009). Similarly, other study in high educated sample showed same results (0.70 and 0.61, respectively; Rubínová et al., 2014). Thus, the discrepancy in these topic could be explained for the differences in educational level of participants and, potentially, other regional features.

### Limitations

We have not included rural populations or with native language other than Spanish. Consequently, the results of this study may not be applicable to these population subgroups. The comparison groups were statistically different for the age, a potential confounding variable. However, we performed a secondary sub-analysis for checking that the performance of the logistic regression model is not affected by the age.

### Conclusion

The psychometric properties of M@T allow its application in subjects with less than 4 years of primary education in urban settings. Cut-off points should be corrected for educational level and, according our data, values of 35 and 26 are useful for distinguishing patients with aMCI and early AD, respectively, in patients with low level of education. However, M@T should not be used in isolation to define dementia, since it measures memory impairment (episodic and semantic) and orientation well, but no other types of cognitive impairment nor functionality. Therefore, the simultaneous use of brief functional tests to compensate for this deficiency is required.

### Recommendations

Recent studies in European populations have evaluated the ability of M@T to discriminate between aMCI and subjective memory complaints (SMC), showing an optimal performance in subjects with medium (Rami et al., 2010) and low educational level (Sousa et al., 2015). Our study did not incorporate this study group. However, we consider that future research should do so because SMC has been reported as a predictor of cognitive decline and AD (Mendonça et al., 2016).

Additionally, the future studies should include population with a broad variability of educational level and higher sample size. Thus, multivariate models could be applied to assess the factors that is statistically associated with clinical diagnosis, which includes the years of education.

The M@T constitutes a brief, non-invasive and reliable cognitive test, which could be applicable for non-specialist physicians to support the discrimination between aMCI and early AD in primary care centers.

## Author Contributions

NC performed the conception of the study. NC, DL, RM and EH-P designed the study. NC, DL, RM, SC-S, JC-A and LV-L collected the data. NC and EH-P analyzed and interpreted the data of the work. EH-P and NC drafted the first draft of the article. All authors critically revised the manuscript and approved the version to be published.

## Conflict of Interest Statement

The authors declare that the research was conducted in the absence of any commercial or financial relationships that could be construed as a potential conflict of interest.
