# Supplementary material for: Memory Alteration Test to Detect Amnestic Mild Cognitive Impairment and Early Alzheimer’s Dementia in Population with Low Educational Level
Source: Front Aging Neurosci. 2017 Aug 22;9:278. doi: 10.3389/fnagi.2017.00278 (PMC5572224; doi:10.3389/fnagi.2017.00278)
Supplement: Supplementary file 1 [file Table_1.docx]

**Additional analysis**

We performed a stepwise analysis for assessing if the score of memory alteration test (M@T) is statistically associated with clinical diagnosis (early Alzheimer´s disease or amnestic mild cognitive impairment). Thus, we built a logit model with clinical diagnosis as the dependent variable and age, gender, years of education, and psychometric test scores (memory alteration tests, Mental State of Examination, and Clock Drowing Test – Mano´s version) as independent variables.

The selection of the variables for the final model were performed by backward method and the significance level for removal from the model was 0.20. We specified that the age and years of education was included in the model and not be subjected to the selection criteria.

| **Table 4.** Multivariate model for assessing the variables statistically associated with clinical diagnosis. | | | | | | |
| --- | --- | --- | --- | --- | --- | --- |
|  |  | Early AD versus aMCI | |  | aMCI versus control | |
|  |  | Odds ratio | Std. Err. |  | Odds ratio | Std. Err. |
|  |  |  |  |  |  |  |
| Age, years | | 0.722 | 0.240 |  | 0.837 | 0.134 |
| Education, years | | 0.554 | 0.412 |  | 0.771 | 0.373 |
| Sex |  | 135.70 | 495.65 |  | EV | EV |
| M@T, score | | 5.162 | 5.215 |  | 6.863* | 5.836 |
| MMSE, score | | 7.525 | 11.374 |  | 5.297 | 5.298 |
| CDT, score | | OV | OV |  | 7.786 | 8.727 |
|  |  |  |  |  |  |  |
|  | | | | | | |
| *AD: Alzhéimer´s dementia; aMCI: amnestic Mild Cognitive Impairment; MMSE: Mini Mental State of Examination; CTD: Clock Drowing Test – Mano´s version; M@T: Memory Alteration Test; OV: omitted variable because predicts data perfectly; EV: excluded variable because p-value >= 0.2; *: p-value < 0.05* | | | | | | |

According results showed in table 4, the score of M@T is associated with a clinical discrimination between aMCI and controls, adjusted by age, years of education, sex and other psychometric tests (MMSE and CDT). However, this adjusted model was not statistically significantly for the discrimination between early AD and aMCI.
